# Transcriptional control of the gonococcal *ompA* gene by the MisR/MisS two-component regulatory system

**DOI:** 10.1038/s41598-020-66382-2

**Published:** 2020-06-10

**Authors:** Concerta L. Holley, Julio C. Ayala, William M. Shafer

**Affiliations:** 1 Department of Microbiology and Immunology, Emory University School of Medicine, Atlanta, GA 30032 USA; 2 The Emory Antibiotic Resistance Center, Emory University School of Medicine, Atlanta, GA 30032 USA; 3 Laboratories of Bacterial Pathogenesis, Veterans Affairs Medical Center, Decatur, GA 30039 USA

**Keywords:** Microbiology, Molecular biology

## Abstract

*Neisseria gonorrhoeae*, the causative agent of gonorrhea, is an exclusive human pathogen whose growing antibiotic resistance is causing worldwide concern. The increasing rise of antibiotic resistance expressed by gonococci highlights the need to find alternative approaches to current gonorrhea treatment such as vaccine development or novel therapeutics. The gonococcal OmpA protein was previously identified as a potential vaccine candidate due to its conservation and stable expression amongst strains of *Neisseria gonorrhoeae*. However, factors that might modulate levels of OmpA and therefore potential vaccine efficacy are unknown. Earlier work indicated that *ompA* is part of the MisR/MisS regulon and suggested that it was a MisR-activated gene. Herein, we confirmed MisR/MisS regulation of *ompA* and report that the MisR response regulator can bind upstream of the *ompA* translational start codon. Further, we describe the contribution of a DNA sequence upstream of the *ompA* promoter that is critical for MisR activation of *ompA* transcription. Our results provide a framework for understanding the transcription of gonococcal *ompA* through a regulatory system known to be important for survival of gonococci during experimental infection.

## Introduction

The strict human pathogen *Neisseria gonorrhoeae* (*Ng*) is the etiologic agent of gonorrhea, which is the second most common sexually transmitted infection in the United States and causes an estimated 87 million infections globally per year^1,2^. Historically, *Ng* has developed clinical resistance to every antibiotic introduced for therapy of gonorrhea^3^. Worryingly, extensively drug-resistant *Ng* strains have been reported globally that are resistant to azithromycin and/or ceftriaxone, which are currently used in dual antibiotic therapy in the United States and elsewhere^4–8^. The current crisis of antibiotic resistance expressed by *Ng* strains and overall reduced industrial efforts to develop new antimicrobial drugs has renewed interest in developing a gonorrhea vaccine^9^. In this respect, several surface-exposed, conserved and stably produced *Ng* antigens have been proposed as vaccine candidates; included in this list is a 23 kDa outer membrane protein termed OmpA that is similar to OmpA in other Gram-negative bacteria^9,10^.

OmpA-like proteins have been considered as vaccine targets^11–15^. For instance, mucosal immunization of mice with purified OmpA elicited protective immunity against multi-drug resistant *Acinetobacter baumannii*^16^. *Ng* OmpA was initially discovered by *in silico* screening of the FA1090 genome database for potentially surface-exposed proteins that could be vaccine antigens for an *Ng* vaccine^10^. As *Ng* OmpA is present and conserved by all examined *Ng* strains and not subject to phase or antigenic variation, it is an ideal target for recognition by the immune system. Relevantly, sera from *Ng-*infected patients recognized OmpA indicating its expression during natural human infection^10,17^. Consideration of OmpA as a vaccine candidate is further supported by findings that it facilitates *Ng* adhesion to and invasion of human cervical and endometrial cells, resistance to phagocytosis and survival during experimental infection of the lower genital tract of female mice^10^. Taken together, these studies implicate OmpA as a virulence factor that could be exploited as part of a vaccine to protect at-risk individuals from gonorrhea.

Notably, *ompA* was shown to be amongst the approximately 17% of *Ng* genes differentially expressed during symptomatic, natural cervical infection in women compared to *Ng* (strain NCCP11945) grown in chemically defined broth^18^. Thus, understanding mechanisms of *Ng* transcriptional control of *ompA* expression could help to advance knowledge regarding the role of OmpA in *Ng* pathogenesis during infection and advance vaccine development efforts. In this respect, there is evidence from studies with other bacteria that *ompA*-like genes are subject to transcriptional control systems. For instance, *ompA* has been reported to be regulated by Hfq and small RNAs such as MicA and SSr1 in *Escherichia coli* and *Shigella flexneri*^19–21^. However, *Ng ompA* does not appear to be regulated by Hfq or any of the predicted *Ng* sRNAs^18,22^. Nevertheless, evidence for transcriptional regulation of *Ng ompA* is suggested by results from two independent transcriptional profiling studies that included *ompA* as a gene that can be activated by the MisR/MisS sensory two-component regulatory system (TCS)^23,24^. MisR/MisS is similar to CpxR/CpxA possessed by other bacteria^25^ and consists of the MisR response regulator and MisS sensory histidine kinase (MisS) responsible for phosphorylation of MisR. Although environmental signals that activate MisR/MisS remain unknown, this TCS was reported to be essential for *Ng* survival during experimental infection of the lower genital tract of female mice^24^. Accordingly, we sought to define the mechanism of MisR regulation of *ompA* and herein provide a model for MisR activation of this *Ng* virulence gene.

## Results and Discussion

### Confirmation of MisR/MisS regulation of *ompA*

**Figure 1:**
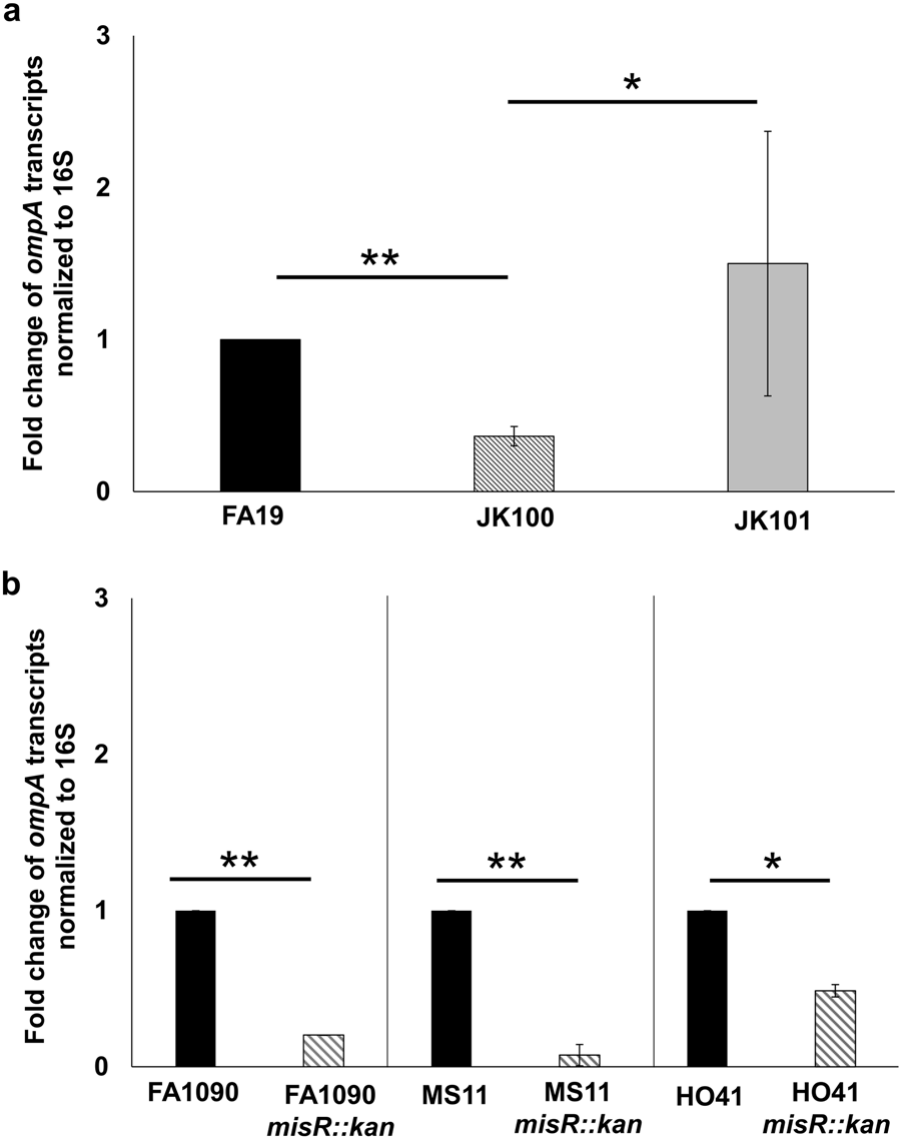
MisR is necessary for expression of *ompA*. (**a**) qRT-PCR analysis of *ompA* transcripts in FA19, *misR-*null (JK100), and complemented strain (JK101) at the mid-logarithmic phase of growth. (**b**) qRT-PCR analysis of *ompA* transcripts in strains FA1090, MS11, and HO41 and their respective *misR-*null mutants. Error bars represent standard deviations from the means of 3 independent experiments. Normalized Expression Ratios (NER) were calculated using 16S rRNA expression. The statistical significance of the results was determined by Student’s t-test, *P < 0.05, **P < 0.001.

Our previous work and that of others that defined the *Ng* MisR/MisS regulon identified *ompA* as being transcriptionally activated by MisR^23,24^. To confirm these observations, we examined *ompA* transcript and protein levels in wild-type (WT) strain FA19, its isogenic *misR*-null mutant (JK100) and complemented strain (JK101) by quantitative reverse transcription-polymerase chain reaction (qRT-PCR) analysis. The results confirmed that *ompA* expression is elevated when MisR is present (Fig. 1) as is the level of OmpA (Supplementary Fig. S1). To ensure that regulation of *ompA* due to loss of MisR is not restricted to the FA19 genetic background, we also examined *ompA* transcript levels in *misR::kan* mutants from other *Ng* strains (e.g., FA1090, MS11, and HO41). Results from qRT-PCR analysis showed that compared to their respective parental strain, the *ompA* transcript level was also reduced in the MisR-negative mutant (Fig. 1b).

In TCSs, the sensor kinase functions to not only sense the environmental stress cues but also to phosphorylate its cognate regulator to enhance the latter’s activity^26,27^. To determine if MisR regulation of *ompA* expression requires the MisS sensory kinase, we examined *ompA* expression in MisS-negative strain JK102. Results from qRT-PCR analysis showed a significant reduction in the *ompA* transcript level due to the loss of MisS (Supplementary Fig. S2), which was reversed by complementation with the WT *misS* gene expressed in *trans*. Thus, both MisR and MisS participate in activation of *ompA* expression.

### MisR directly regulates *ompA* expression

**Figure 2:**
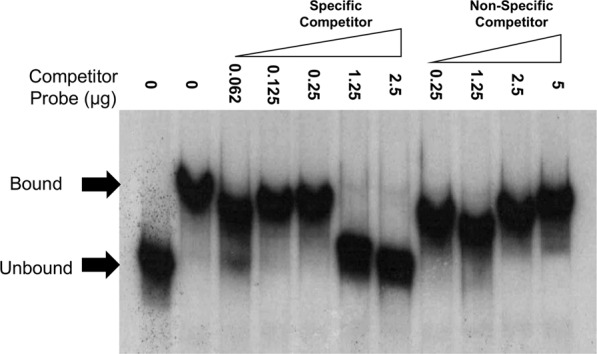
MisR binds to the *ompA* promoter in a specific manner. Shown is a competitive EMSA demonstrating MisR binding specificity to the *ompA* promoter. Lane 1, radiolabeled probe alone (5 ng); lane 2, radiolabeled probe plus MisR~P (1.5 µg); lanes 3–7, radiolabeled probe plus increasing concentrations of the unlabeled *ompA* probe (specific); lanes 8–11, radiolabeled probe plus increasing concentrations of the unlabeled *rnpB* probe (non-specific).

We next determined if MisR regulation of *ompA* is direct. For this purpose, an electrophoretic mobility shift assay (EMSA) was performed using *in vitro* phosphorylated MisR (MisR~P) and 400 bp of the DNA sequence upstream of the *ompA* translational start codon that contains putative promoter elements. The results showed that MisR~P could bind to the target DNA (Fig. 2 and Supplemental Fig. 3). As a control, we determined if another gonococcal transcriptional regulatory protein, MpeR, could bind the examined *ompA* promoter. The regulon of MpeR has some overlap with genes regulated by MisR/MisS^28^ but does not regulate *ompA*. We confirmed that MpeR does not bind *ompA* target DNA, suggesting that the interaction between *ompA* and MisR is specific (Supplemental Fig. S4).

To ensure that MisR~P binding to the target DNA was specific, we performed a competitive EMSA using unlabeled specific (*ompA)* and non-specific *(rnpB*) probes; *rnpB* is not regulated by MisR^23^. Importantly, only the specific unlabeled probe could compete with the labeled *ompA* probe for MisR-binding (Fig. 2) indicating that such binding was specific.

### Identification of MisR target sites upstream of *ompA*

**Figure 3:**
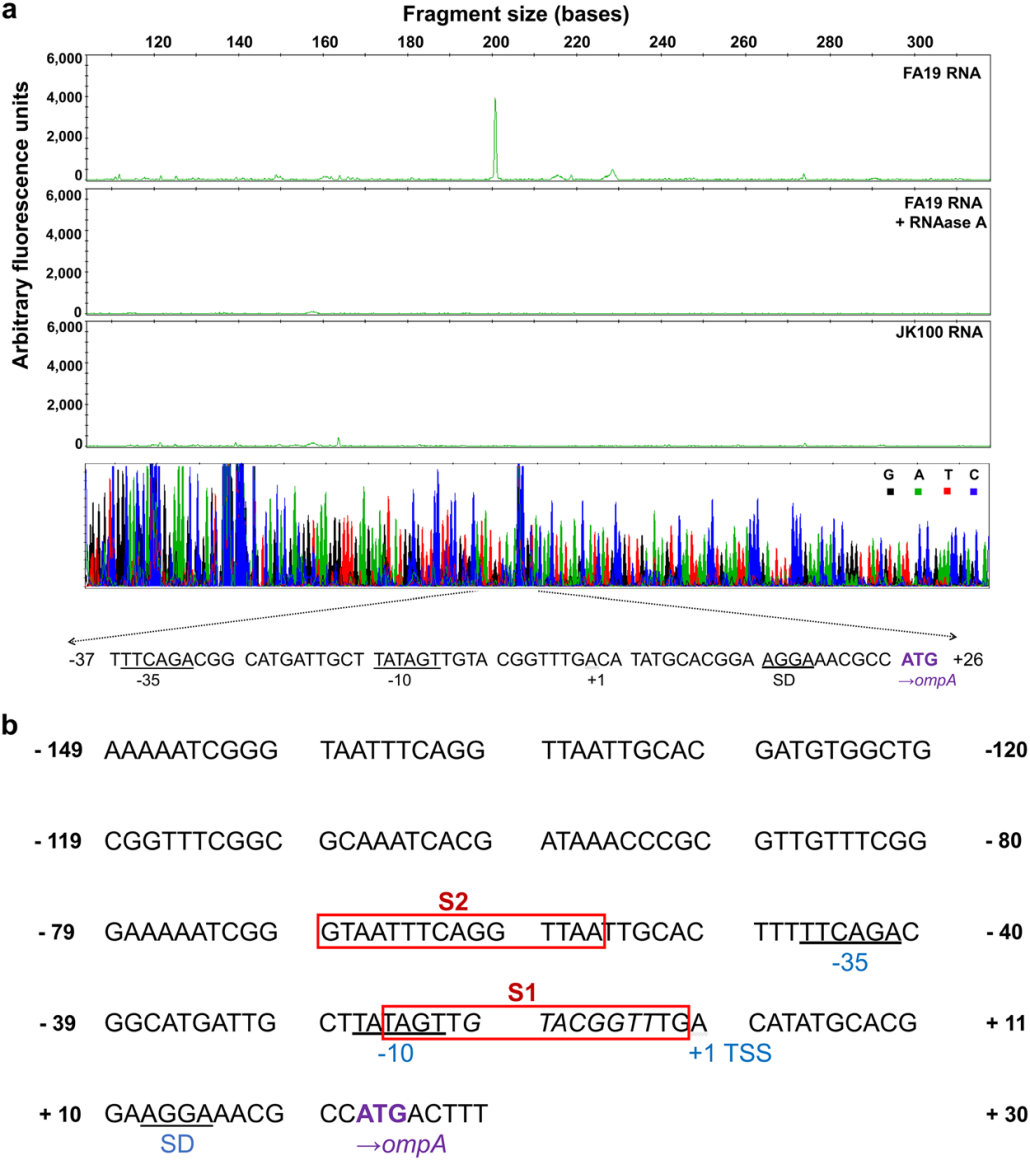
Mapping of transcriptional start sites in the *ompA* promoter by primer extension. (**a**) Electropherograms of fluorescently (HEX) labeled primer extension products for FA19, RNase treated FA19 and *misR-*null (JK100) and the corresponding sequence ladder. The nucleotides shown below the sequence ladder correspond to the promoter region of *ompA*. Primer extension products were analyzed using Applied Biosystems GeneMapper Software version 4.0 (https://www.thermofisher.com/order/catalog/product/4440915#/4440915). (**b**) Sequence of the *ompA* promoter region with predicted MisR-binding sites. Red boxes indicate predicted MisR-binding sites using the PRODORIC online tool. The −10, −35 and Shine-Dalgarno promoter elements and the transcriptional start site (TSS) are indicated with text and underlined. The ATG *ompA* start codon is highlighted in purple. MisR binding site consensus sequence IUPAC code: KWWWTGTAARGNNWH where K = G/T, W = A/T, R = A/G, H = A/T/C, and N = any nucleotide. The nucleotide sequence within the S1 site that was mutated in TruncΔS1 is shown in italics.

After confirmation of MisR binding to the upstream *ompA* DNA, we sought to identify the *ompA* promoter and MisR-binding sites important for the regulation of *ompA* expression. Accordingly, we first mapped the *ompA* promoter by identifying the transcriptional start site (TSS) by primer extension (PE) analysis using total RNA isolated from strains FA19 and JK100 (FA19 *misR::kan*). The PE assay protocol generated a single peak from the FA19 RNA that was absent when such RNA was treated with RNaseA or when RNA from JK100 was used. The PE product obtained with FA19 RNA was positioned approximately 23 bp upstream of the ATG start codon and 11 bp downstream of a −10 sequence of the putative sigma 70 promoter element (Fig. 3a, top panel). Interestingly, the separation of the predicted −10 and −35 hexamers of the putative *ompA* promoter is a sub-optimal 13 nucleotides, which suggests that transcriptional factors (e.g., MisR) are needed for *ompA* transcription.

**Figure 4:**
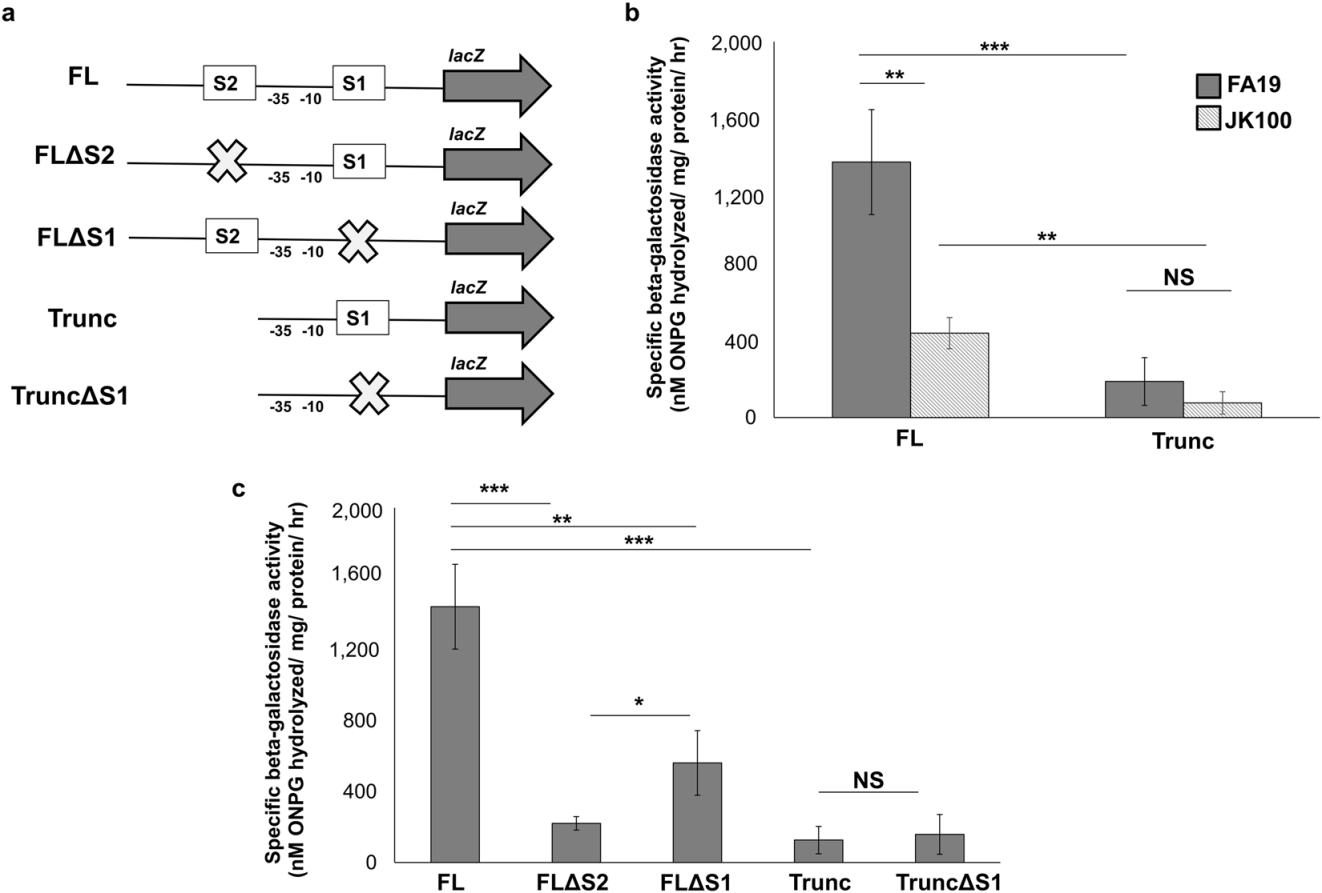
Regulation of *ompA* by MisR. (**a**) The organization of the *ompA-*lacZ fusion constructs are depicted. The approximate locations of the predicted MisR-binding sites are indicated by boxes S1-S2. The −10 and −35 hexamers are notated. (**b**) Regulatory effect of the *misR* mutation on the expression of *ompA*. The specific β-galactosidase activity per mg of total protein in cell extracts of reporter strains containing the *ompA-lacZ* fusions (FL and Trunc) in the FA19 and *misR* null (JK100) backgrounds. (**c**) Effect of disruption of MisR binding sites on the expression of *ompA*. The specific β-galactosidase activity per mg of total protein in cell extracts of reporter strains containing the *ompA-lacZ* fusions (FL and Trunc) and the disrupted MisR binding site fusions (FLΔS2, FLΔS1, and TruncΔS1) in the FA19 background. Results are the average of three independent experiments. Statistical significance was determined by ANOVA, *P < 0.05, **P < 0.01, ***P < 0.001.

Next, we performed an analysis of the putative *ompA* promoter region (illustrated in Fig. 3b) in order to understand the mechanism of MisR regulation. Initially, we used DNaseI protection to identify MisR-binding sites, but the results were inconclusive (data not shown). Consequently, we employed a combination of bioinformatic and genetic studies to identify DNA sequences that might participate in MisR regulation of *ompA*. For bioinformatic analysis, the PRODORIC online tool^29^ was used to identify potential MisR-binding sites upstream of the *ompA* start codon using the previously published *Ng* MisR binding consensus sequence IUPAC code: KWWWTGTAARGNNWH where K = G/T, W = A/T, R = A/G, H = A/T/C, and N = any nucleotide^23^. This analysis suggested the presence of two potential MisR-binding sites that hereafter are referred to as S1 and S2 (Fig. 3b). To determine the significance of the two putative MisR-binding sites, we examined *ompA* expression in WT and *misR::kan* background strains using *ompA*-*lacZ* translational fusions that consisted of both S1 and S2 (full-length fusion, [FL]) or just S1 (truncated fusion [Trunc]) (Fig. 4a). With these *ompA::lacZ* fusion strains, we found that there was a decreased expression of the FL fusion in the *misR-*null strain (JK100) compared to WT strain FA19 indicating that MisR interaction with the *ompA* promoter containing region is essential for WT levels of *ompA* expression (Fig. 4b). Importantly, the presence of only the S1 site in the Trunc fusion resulted in significantly reduced expression compared to the FL fusion in both the WT and *misR::kan* backgrounds (Fig. 4b).

**Figure 5:**
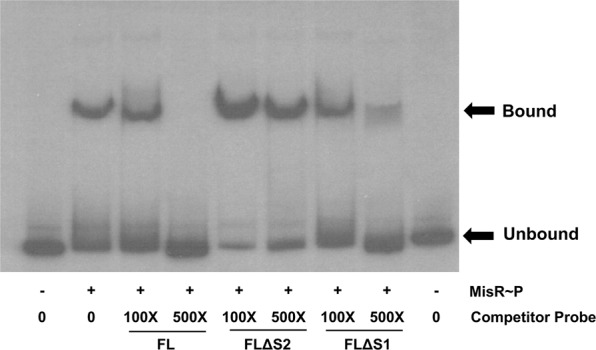
Mapping of primary MisR binding sites. Competitive EMSA demonstrating preferential MisR binding to specific sites in the promoter region. Lane 1 and 9, radiolabeled probe alone (5 ng); Lane 2, radiolabeled probe plus MisR~P (1.5 µg); Lanes 3–4, radiolabeled probe plus unlabeled FL competitor probes; Lanes 5–6 radiolabeled probe plus unlabeled FLΔS2 competitor probes; Lanes 7–8 radiolabeled probe plus unlabeled FLΔS1 competitor probes.

To further assess the contribution of S1 and S2 with respect to MisR control of *ompA* expression, we constructed additional *ompA-lacZ* fusions in WT strain FA19 that had mutations in each site (Fig. 4a). Thus, we deleted the entire S2 site (14 bp) to create FLΔS2. Given that the S1 site overlaps the putative −10 promoter element, we removed 8 bp of the binding site (5′-GTACGGTT-3′) and inserted 8 bp of non-consensus sequence (5′-ACCTTCAC-3′) to create FLΔS1 and TruncΔS1; the region of the sequence changed in the S1 site is shown in italics in Fig. 3b. This fusion construct allowed for loss of the S1 binding site while maintaining the integrity of the −10 element and the TSS. With these fusion strains, we noted an 85% decrease in *ompA* expression when the S2 site was removed from the FL fusion (Fig. 4c). By comparison, disruption of the S1 site reduced expression of the *ompA* promoter to a lesser extent (ca. 50%) (Fig. 4c). Further, there was no significant difference in *ompA-lacZ* expression between the Trunc or TruncΔS1 fusions, although there was still a significant reduction compared to the FL fusion containing both S1 and S2. Thus, although both putative MisR-binding sites may contribute to regulating *ompA* expression, the results suggested that S2 plays a more predominant role in interactions with MisR. To test this hypothesis, we performed a competitive EMSA using the disrupted S1 or S2 site DNAs as unlabeled competitors to the radiolabeled FL probe. As expected, the unlabeled FL probe competed with the radiolabeled FL probe (Fig. 5 and Supplemental Fig. 5) as did the FLΔS1 probe, albeit to a lesser extent. Consistent with the *lacZ* fusion data, the FLΔS2 probe did not compete with the FL probe. Thus, we concluded that the S2 site is required, but not sufficient, for full MisR activation of *ompA* expression in *Ng*.

This work was stimulated by previous observations that collectively suggested important roles for both OmpA and MisR/MisS in the ability of *Ng* to survive during experimental lower genital tract infection of female mice^10,24^. Since *ompA* expression was found in two different studies to be part of the MisR regulon^23,24^, we sought to define the mechanistic basis for MisR/MisS regulation of *ompA* expression. The results presented herein indicate a direct role for MisR control of *ompA*. We propose that while both the S1 and S2 putative MisR-binding sites (Fig. 3b) participate in MisR activation of *ompA* expression the latter plays a more predominant role in this regulation. The location of the S2 site immediately upstream of the −35 hexamer suggests that bound MisR assists recruitment of RNAP to the promoter, which has a sub-optimal spacing between the −10 and −35 hexamers, for transcription of *ompA*. However, we cannot discount a role for the downstream S1 site as disruption of it in WT strain FA19 significantly reduced *ompA* expression even when S2 was present (Fig. 4c).

In conclusion, this is the first report that characterizes regulation of the *Ng* o*mpA* gene, which encodes a candidate vaccine antigen. We propose that MisR/MisS directly enhances ompA expression. Taken together, the intrinsic linkage of MisR/MisS and OmpA could be exploited for vaccine or chemotherapeutic development purposes.

## Methods

### Bacterial strains, plasmids, and primers

*Ng* strain FA19 and its isogenic mutant strains, along with the plasmids used and their *Escherichia coli* hosts, are listed in Table 1. The oligonucleotide primers used in this study are listed in Supplementary Table S1. *E. coli* strains were routinely cultured on Luria-Bertani (LB) agar or in LB broth (Difco, Sparks, MD) containing 50 µg/mL kanamycin, 100 μg/mL ampicillin or 100 μg/mL chloramphenicol as necessary. Gonococci were grown on gonococcal base (GCB) agar (Difco, Sparks, MD) containing Kellogg’s supplements I and II at 37 °C under 5.0% (v/v) CO_2_^30^. Liquid cultures of gonococci were begun by inoculating plate-grown cells in pre-warmed GCB broth containing Kellogg’s supplements I and II and 0.043% (w/v) sodium bicarbonate and grown at 37 °C with shaking. Liquid cultures of gonococci contained a final concentration of 10 mM MgCl_2_.

**Table 1:** Bacterial Strains and plasmids used in this study.

Strain or plasmid	Genotype or description	Reference or source
***N. gonorrhoeae***		
FA19	WT strain	^38^
CH10	FA19 *ompA::ermC*	This Study
CH11	CH10 complementation	This Study
JK100	FA19 *misR::kan*	^23^
JK101	JK100 complementation (FA19 *misR*::*kan*/pGCC4-*misR*)	^23^
FA1090	WT strain	^39^
FA1090 Δ*ompA*	FA1090 *ompA::ermC*	^10^
FA1090 Δ*ompA* C’	FA1090 Δ*ompA* complementation	^10^
FA1090 *misR::kan*	FA1090 *misR::kan*	^23^
MS11	WT strain	^40^
MS11 *misR::kan*	MS11 *misR::kan*	This Study
HO41	WT strain	^41^
HO41 *misR::kan*	HO41 *misR::kan*	This Study
FA19::P_*FL*_	FA19 containing a translational fusion of 123 bp of the promoter region of *ompA* to the *lacZ* gene	This Study
FA19::P_*FLΔS1*_	FA19 containing a translational fusion of 123 bp of the promoter region of *ompA* to the *lacZ* gene and S1 disruption	This Study
FA19::P_*FLΔS2*_	FA19 containing a translational fusion of 123 bp of the promoter region of *ompA* to the *lacZ* gene and S2 deletion	This Study
FA19::P_*Trunc*_	FA19 containing a translational fusion of 81 bp of the promoter region of *ompA* to the *lacZ* gene	This Study
FA19::P_*TruncΔS1*_	FA19 containing a translational fusion of 81 bp of the promoter region of *ompA* to the *lacZ* gene and S1 disruption	This Study
***Escherichia coli***		
One Shot TOP10	F^−^ *mcr*A Δ(*mrr-hsdRMS-mcrBC*) ϕ80*lacZ*ΔM15 Δ*lacX74 recA1 araD139* (*ara leu*)*7697 galU galK rpsL* (Str^r^) *endA1 nupG*	Invitrogen (Carlsbad, CA)
BL21(DE3)	*fhuA2 [lon] ompT gal (λ DE3) [dcm] ΔhsdS λ*	New England Biolabs (Ipswitch, MA)
**Plasmids**		
pET-15b	Bacterial expression vector with T7lac promoter, N-terminal His-tag	Merck Millipore (Burlington, MA)
pCH1	pET-15b containing FA19 *ompA* coding region	This Study
pLES94	pUC18 derivative containing a truncated *lacZ* gene for use in translational fusions; recombines at the *proAB* locus of the gonococcal chromosome	^36^
pCH22	pLES94 containing 123 bp upstream of *ompA* (FL)	This Study
pCH23	pLES94 containing 81 bp upstream of *ompA* (Trunc)	This Study
pCH24	pCH22 with disrupted S1 site	This Study
pCH25	pCH22 with deleted S2 site	This Study
pCH26	pCH23 with disrupted S1 site	This Study

### Generation of *ompA* and *misR*-null mutants

Construction of the FA19 *ompA::ermC* mutant (strain CH10) was performed as described below using an erythromycin resistance cassette^10^. CH10 was constructed by transforming WT FA19 with a purified PCR product made from genomic DNA from the *Ng* FA1090 *ompA::ermC* mutant constructed previously^10^ and generously donated by Ann Jerse (Uniform Services University, Bethesda, MD). Plate transformations were performed as described previously and transformants selected on GCB agar containing erythromycin 1 μg/mL^31^. Insertion of the *ermC* cassette was confirmed by PCR using primers HFLF2 and HFLR2 and verified by sequencing of PCR product made from genomic DNA. *misR::kan* mutants in strains FA1090, HO41 and MS11 were constructed by inactivating the *misR* gene using the nonpolar *aphA-3* kanamycin cassette as described previously^23^. Loss of *misR* was confirmed by PCR and sequencing using primers misRkanup and misRkandown.

### Complementation of the *ompA::ermC* mutant

*Ng* strain CH10 was complemented as follows. In FA1090 *ompA::ermC C’*, the entire *ompA* gene and its native promoter are inserted into an intergenic region in the chromosome between NGO0077 and NGO0078^10,32^. The complemented coding region was amplified using primers PNG0077 and PNG0078 to ensure recombination of the complement in the correct locus. Transformants of CH10 were selected on GCB agar using chloramphenicol 10 μg/mL and verified by PCR and sequencing.

### qRT-PCR analysis of Ng transcripts

For measurement of target gene expression, gonococci were harvested at mid- or late-log phase and the pellets were stored at −70 °C. RNA was purified by Trizol extraction as per manufacturer instructions (Thermo Fisher Scientific, Waltham, MA) followed by Turbo DNA-free (Ambion, Austin, TX) treatment. cDNA was generated using a QuantiTect reverse transcriptase kit (Qiagen, Venlo, Netherlands). We validated our qRT-PCR methods by examining primer efficiency, primer specificity (melt temperature) and linear dynamic range for each primer pair utilized herein. For additional information about our validation results, see Supplemental Fig. S6. For qRT-PCR analysis, the normalized expression of each target gene was calculated using 16 S rRNA as a housekeeping reference gene^33^. As an additional internal control, significance was confirmed using *recA* as the reference gene (data not shown). All qRT-PCRs were performed in technical and biological triplicates.

### Purification of recombinant His-OmpA protein and preparation of polyclonal antisera

The coding sequence of *ompA* was amplified with primers His-OmpAF and His-OmpAR. The PCR product was digested with BamHI and XhoI and then cloned into pET-15b which had been digested with the same enzymes to yield pCH1. The plasmid was purified and transformed into *E. coli* expression strain BL21(DE3). A His-OmpA fusion protein was produced using a hybrid purification method denaturing the protein to enable solubilization first and renaturing the protein on the column prior to elution as per manufacturer’s protocol (Millipore Sigma, Burlington, MA). The fusion protein was purified using a nickel-nitrilotriacetic acid (Ni^+2^ -NTA) column. His-OmpA was eluted with buffer containing 100 and 200 mM imidazole. The fractions were dialyzed to remove imidazole using 10 mM PBS (137 mM NaCl, 2.7 mM KCl, 10 mM Na_2_HPO_4,_ 2 mM KH_2_PO_4_) and concentrated. Dithiothreitol (DTT) and glycerol were added to a final concentration of 1 mM and 10% (v/v), respectively. The purity of recombinant OmpA was confirmed by SDS‐PAGE electrophoresis staining with Coomassie blue. A rabbit polyclonal anti-OmpA antibody was generated (Pacific Immunology, Ramona, CA) using a small peptide corresponding to amino acids 145–159 of the OmpA protein (Cys-NGHTDNTGSDAVNNP). The specificity of the antibody was tested against whole cell lysates purified from FA19 and *ompA* mutant strains as well as the purified His-OmpA protein.

### Western blotting

Gonococci grown to late-log phase in broth were pelleted by centrifugation at 10,000 rpm for 2 min, and whole-cell lysates were prepared in 2X SDS loading dye (100 mM Tris-HCl, pH 6.8, 4% [wt/vol] SDS, 0.2% [wt/vol] bromophenol blue, 20% glycerol, 200 mM dithiothreitol [DTT]). Protein levels were normalized by use of a NanoDrop spectrophotometer and BCA Protein Assay. Equivalent loading was confirmed by Coomassie staining on a 12% SDS-polyacrylamide gel. Blots were blocked in 5% (wt/vol) nonfat dried milk in 1X TST buffer (0.01 M Trizma base, 0.150 M NaCl, 0.05% [vol/vol] Tween 20) and probed with primary antibody against OmpA O/N at 4 °C using a 1:1000 dilution. Blots were then washed with 1X TST before incubation with secondary antibody conjugated to AP and developed with NBT/BCIP.

### EMSA for detection of MisR binding to target DNA

A DNA probe containing the putative *ompA* promoter region (Fig. 3b) was amplified by PCR from FA19 genomic DNA using the primers pOmpA2F and pOmpAR. For radiolabeled probes, the indicated PCR product was labeled with [γ^32^P]-dATP using T4 polynucleotide kinase (New England Biolabs, Ipswitch, MA). The labeled DNA fragments (5 ng) were incubated with 1.5 μg of MpeR and MisR that had been phosphorylated with acetyl phosphate in a 30 μl reaction buffer at room temperature^34^. For MisR competition assays, the unlabeled *ompA* probe or an unlabeled PCR product (5 ng) using RnpB1F and RnpB1R primers (non-specific *rnpB* probe) were incubated with protein for 15 minutes prior to the addition of the radiolabeled probe. Samples were subjected to electrophoresis in a 6% native polyacrylamide gel at 4 °C, followed by autoradiography.

### Primer extension analysis

The *ompA* TSS was identified by primer extension using a 5′-fluorescently labeled HEX primer and analysis on an automated capillary electrophoresis instrument as described previously^35^ with modifications. FA19 and JK100 were grown to an OD_600_ of 1.0, and 1 mL of the culture was resuspended in 200 μl of RNAlater solution (Ambion, Austin, TX). Total RNA was isolated by using the RNeasy Plus Minikit (Qiagen, Venlo, Netherlands), contamination with genomic DNA was removed using the Turbo DNA-free Kit (Invitrogen, Carlsbad, CA), and DNase I-digested total RNA samples were further concentrated with the Qiagen RNeasy MiniElute Cleanup kit (Qiagen, Venlo, Netherlands). Twenty-seven µg of total RNA was annealed with 30 picomoles of primer Hex-Om-IT-R in a thermocycler using the program 90 °C for 3 min, 53 °C (Tm) for 1 hour and 25 °C for 10 min. Then, it was extended for 1 h at 42 °C using the SuperScript II Reverse Transcriptase system (Invitrogen, Carlsbad, CA) following the company protocol. Two µl of the primer extension reactions were combined with 7.5 μl Hi-Di formamide and 0.5 μl GeneScan 600 LIZ size standard (Applied Biosystems, Waltham, MA) and detected with a 3730 capillary DNA analyzer (Applied Biosystems, Waltham, MA) running a default genotyping module. The length and abundance (height and area below the peaks) of the HEX-labeled cDNA primer extension products were analyzed by using Applied Biosystems GeneMapper Software version 4.0 (https://www.thermofisher.com/order/catalog/product/4440915#/4440915).

To accurately assign a nucleotide base to the peaks detected in the primer extension reaction, a sequence ladder was generated by using the Thermo Sequenase Cycle Sequencing Kit (Applied Biosystems, Waltham, MA). Briefly, a DNA template was generated by PCR using primers OmLacFL and Om-It-R2. Sequencing reactions were conducted with 200 fmol of template DNA and 2 pmol of Hex-Om-IT-R (template strand) according to the manufacturer’s instructions. Each of the four dideoxy reaction mixtures was diluted 5-fold in water, and 2 μl was loaded onto the 3730 DNA analyzer (Applied Biosystems, Waltham, MA). The electropherograms of the sequencing reactions were horizontally aligned with those generated in the primer extension using GeneMapper 4.0 (Applied Biosystems, Waltham, MA).

### Construction of the *ompA-lacZ* fusions

*ompA-lacZ* translational fusions were generated using the pLES94 system^36^. Briefly, putative promoter regions of *ompA* were amplified using primers OmLac21, OmLac1 and OmLacRev and used to generate translational fusions of *ompA* to the truncated, promoter-less *lacZ* gene in pLES94. The constructs were transformed into One Shot TOP10 chemically competent *E. coli* cells (Invitrogen, Carlsbad, CA) by heat shock, and transformants were selected on LB agar containing 100 μg/mL ampicillin and 40 μg/mL X-Gal (5-Bromo-4-chloro-3-indolyl-β-D-galactopyranoside). The plasmids were confirmed, purified, and transformed into the wild type strain FA19 to generate strains FA19::P_*FL*_ *and* FA19::P_*Trunc*_. Gonococcal transformants were selected on GCB agar containing 1 μg/mL of chloramphenicol and further verified by PCR.

For the construction of the disrupted site fusions, the suspected binding sites were deleted (ΔS2) or altered (ΔS1) using primers S2disF/R and S1disF/R respectively. The full-length promoter was amplified from a PCR product containing the disruptions and cloned into pLES94. The resulting plasmids were transformed into FA19 to generate strains FA19::P_*FLΔS2*_, FA19::P_*FLΔS1*_, FA19::P_*TruncΔS1*_. *misR*-null strains were constructed by deleting *misR* in the WT fusions strains. All strains were confirmed by sequencing.

### Preparation of cell extracts and β-galactosidase assays

*Ng* strains containing *lacZ* translational fusions were grown overnight on GCB agar plates containing 1 μg/mL of chloramphenicol. Cells were scraped, washed once with phosphate-buffered saline (pH 7.4), and resuspended in lysis buffer (0.25 mM Tris [pH 8.0]). Cells were then broken by three freeze-thaw cycles. The cell debris was removed by centrifugation at 15,000 × *g* for 10 min at 4 °C. β-Galactosidase assays were performed as previously described^37^.

### Statistical methods

All the data were expressed as means with standard deviation (SD). Statistical significance between all quantitative data are analyzed by Student *t*-tests or one-way ANOVA followed by Tukey’s honestly significant difference post-hoc test. Significance was set at *P* < 0.05.

## Supplementary information

Supplementary information.

## Data Availability

The datasets that supported the findings of this study are available from the corresponding author upon request.
