# Supplementary material for: Transcriptional control of the gonococcal ompA gene by the MisR/MisS two-component regulatory system
Source: Sci Rep. 2020 Jun 10;10:9425. doi: 10.1038/s41598-020-66382-2 (PMC7286886; doi:10.1038/s41598-020-66382-2)
Supplement: Supplementary file 1 — Supplementary information. [file 41598_2020_66382_MOESM1_ESM.pdf]

SUPPLEMENTARY INFORMATION

Transcriptional control of the gonococcal *ompA* gene by the MisR/MisS two-component regulatory system

Concerta L. Holley[1], Julio C. Ayala[1], and William M. Shafer[1,2,3*]

[1]Department of Microbiology and Immunology and [2]The Emory Antibiotic Resistance Center, Emory University School of Medicine, Atlanta, GA, 30032, USA and

[3]Laboratories of Bacterial Pathogenesis, Veterans Affairs Medical Center, Decatur, GA, 30039, USA

CONTENTS

Supplementary Table S1: Primers used in this study

Supplementary Table S2: Bacterial Strains and plasmids used in this study (Supplemental Figures Only)

Supplementary Figure S1: MisR is necessary for expression of OmpA

Supplementary Figure S2: MisS and MisR are necessary for expression of *ompA*

Supplementary Figure S3: MisR binds to the *ompA* promoter in a specific manner (Full autoradiograph)

Supplementary Figure S4: MisR and not MpeR binds to the *ompA* promoter

Supplementary Figure S5: Mapping of primary MisR binding sites (Full autoradiograph)

Supplementary Figure S6: Validation of qPCR Method

Supplementary Table S1 - Primers used in this study

Primer Name	Sequence (5’ to 3’) †	Purpose
HFLF2	TATATAGGATCCTATATTAATTAAATGACTTTCTTCAAACCC	Confirmation of *ompA* mutants
HFLR2	TATATACTCGAGTATAGTTTAAACTTACATGTGCCGTGCGGC GTT	Confirmation of *ompA* mutants
pOmp2F	CCGCCTTAGCTCAAAGAGAA	Construction of *ompA* EMSA probe
pOmpAR	TTAAGAATTCCGCCACCCAAACCGTACAT	Construction of *ompA* EMSA probe
RnpB1F	CGGGACGGGCAGACAGTCGC	EMSA control probe
RnpB1R	GGACAGGCGGTAAGCCGGGTTC	EMSA control probe
Hex-Om-IT-R	[HEX]AATTGCGTGCGCCTTTGC	Primer Extension
OmLacFL	TTGGATCCAGTGAATCGGTTCCGTACTATATCTGTACTGT	Primer Extension
Om-It-R2	GCAGTGTTCAGGGCGTATT	Primer Extension
His-OmpAF	TATACTCGAGATGACTTTCTTCAAACCCTTT	Construction of pCH1
His-OmpAR	TATAGGATCCTTACATGTGCCGTGCGGCGTT	Construction of pCH1
misRkanup	GCAAACTGCCCGTATGGGCA	Confirmation of *misR* deletion
misRkandown	GTTGTTGGCGGCAAACAGCC	Confirmation of *misR* deletion
16Smai-RTF	CCATCGGTATTCCTCCACATCTCT	qRT-PCR (reference gene)
16Smai-RTR	CGTAGGGTGCGAGCGTTAATC	qRT-PCR (reference gene)
ompA qRTF	CCCAAACAAATCCGCCATGT	qRT-PCR
ompA qRTR	CGCTGCTCTTGGTAGTCCATAT	qRT-PCR
misR qRT F	TGAGCGGGCAATACGATGTA	qRT-PCR
misR qRT R	ACCCATTTCCAAGCCGATGA	qRT-PCR
OmLac21	TTGGATCCGATAAACCCGCGTTGTTTCGGGAA	Construction of pCH22 (forward)
OmLac1	TTGGATCCTTAATTGCACTTTTTCAGACG	Construction of pCH23 (forward)
OmLacRev	TTGGATCC**TT**GAAAGTCATGGCGTTTCCTT	Construction of ples94 fusions (reverse)
lacZrev	ACGACGACAGTATCGGCCTCAGAA	Confirmation of pLES94 fusion constructs
PNGO0077	TCCCCCTCGATTTGGTGGCACAACA	Complementation of *ompA*
PNGO0078	ACGGGCTGCGCATCATGGCAAACCA	Complementation of *ompA*
S1disF	TATAGTTACCTTCACTGACATATG	Construction of disrupted S1 fusion
S1disR	CATATGTCAGTGAAGGTAACTATA	Construction of disrupted S1 fusion
S2disF	AATCGGGTAGCACTTTTTCAGACG	Construction of disrupted S2 fusion
S2disR	CGTCTGAAAAAGTGCTACCCGATT	Construction of disrupted S2 fusion

† Restriction endonuclease cut sites are underlined.
Bold indicates the addition of a partial 3rd codon to prevent frameshift after BamHI cleavage.

Supplementary Table 2 - Bacterial Strains and plasmids used in this study (Supplemental Figures Only)

Strain or plasmid	Genotype or description	Reference or source
N. gonorrhoeae		
FA19	WT strain	[1]
JK102	FA19 *misS::kan*	[2]
JK103	JK102 complementation (FA19 *misS*::*kan*/pGCC4-*misS*)	[2]

1 Sarubbi, F. A., Jr., Blackman, E. & Sparling, P. F. Genetic mapping of linked antibiotic resistance loci in *Neisseria gonorrhoeae*. *J Bacteriol* **120**, 1284-1292, https://jb.asm.org/content/120/3/1284 (1974).

2 Kandler, J. L. *et al.* The MisR Response Regulator Is Necessary for Intrinsic Cationic Antimicrobial Peptide and Aminoglycoside Resistance in *Neisseria gonorrhoeae*. *Antimicrob Agents Chemother* **60**, 4690-4700, DOI:10.1128/AAC.00823-16 (2016).

Figure S1

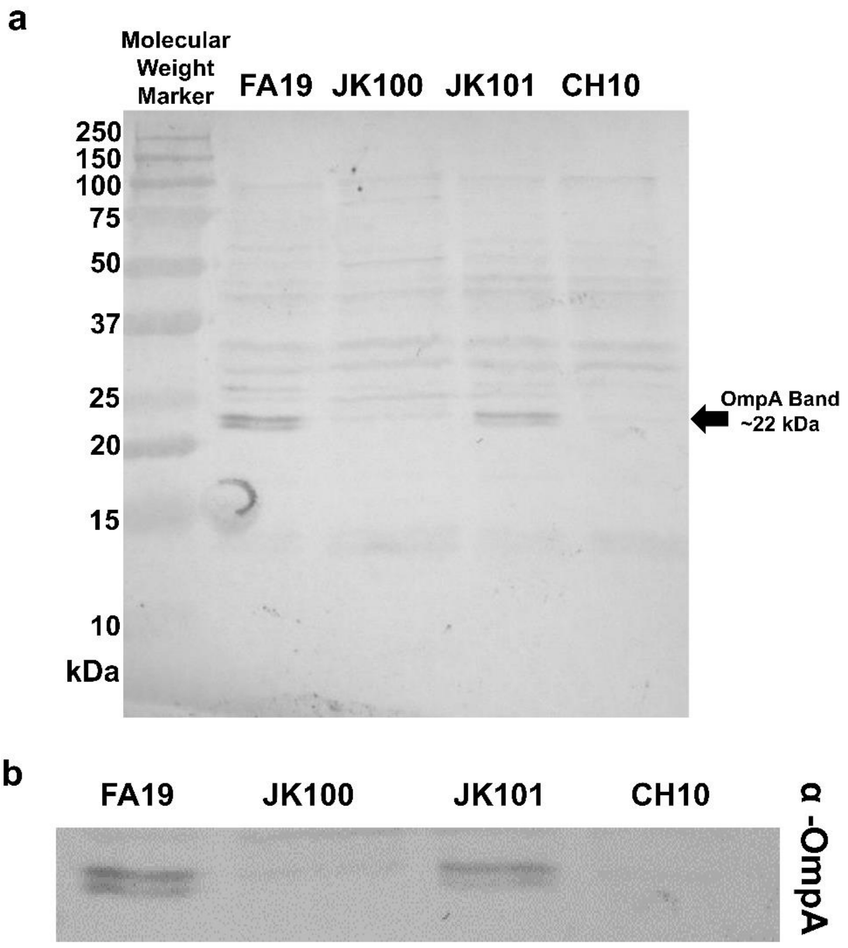

MisR is necessary for expression of OmpA. A - Shown is the full Western blot of 5 µgs of SDS-PAGE separated protein from whole cell lysates of isogenic *Ng* strains FA19 (WT), JK100 (*misR::kan*), JK101 (JK100 *misR*$^{+}$) and CH10 (*ompA::ermC*). The blot was probed with rabbit polyclonal anti-OmpA antiserum as described in Materials and Methods. The position of the OmpA band is indicated by the arrow and text. **B -** Enlargement from Panel A showing close-up of the OmpA protein containing region.

Figure S2

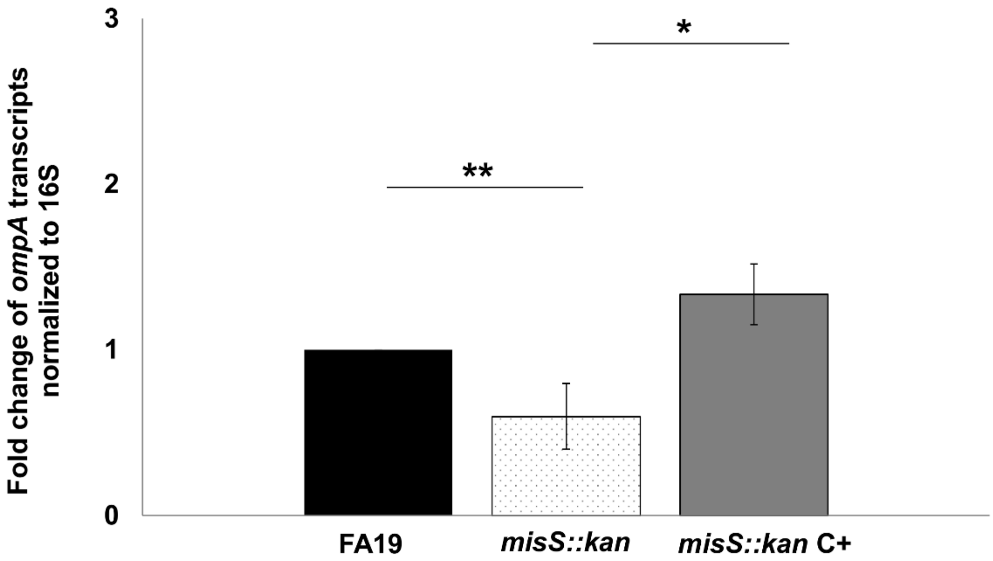

MisS and MisR are necessary for expression of *ompA*. A - qRT-PCR analysis of *ompA* transcripts in FA19, *misS*-null (JK102), and complemented strain (JK103) at the mid-logarithmic phase of growth. Error bars represent standard deviations from the means of 3 independent experiments. Normalized Expression Ratios (NER) were calculated using 16S rRNA expression. The statistical significance of the results was determined by one-way ANOVA followed by Tukey's honestly significant difference post-hoc test. * = $P < 0.05$, ** = $P < 0.001$

Figure S3

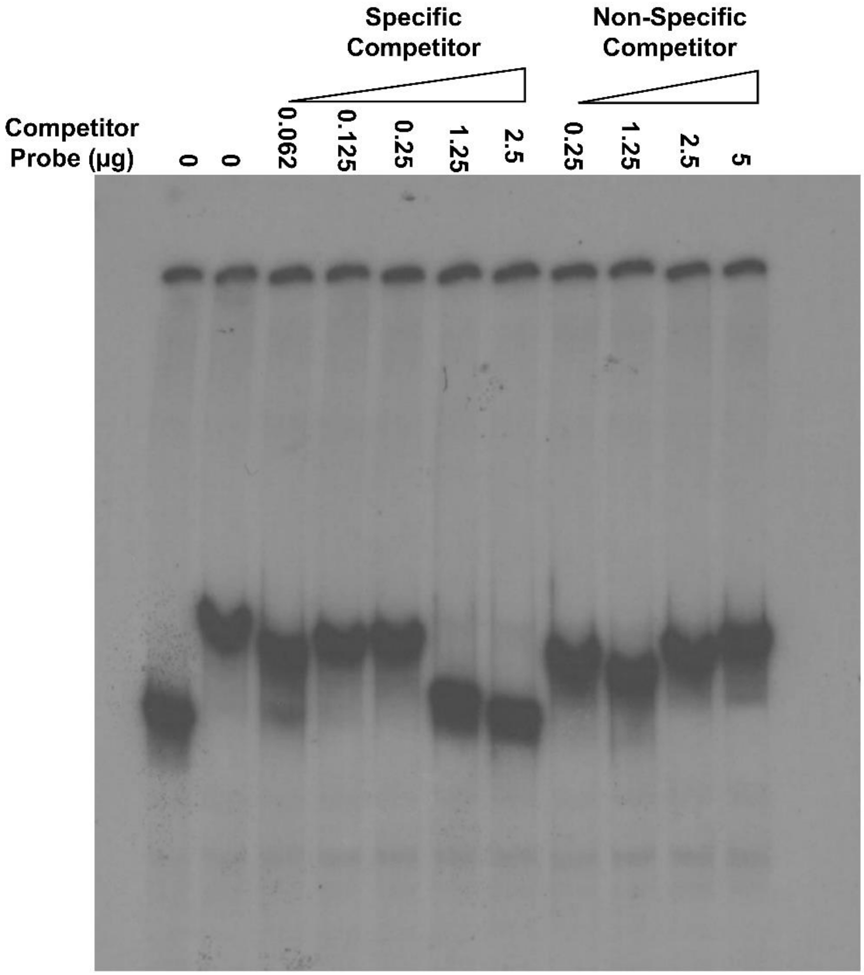

MisR binds to the *ompA* promoter in a specific manner (Full Autoradiograph). Shown is the full-length autoradiograph for the competitive EMSA shown in Figure 2 demonstrating MisR binding specificity to the *ompA* promoter. Lane 1, radiolabeled probe alone (5 ng); lane 2, radiolabeled probe plus MisR~P (1.5 µg); lanes 3-7, radiolabeled probe plus increasing concentrations of the unlabeled *ompA* probe (specific); lanes 8-11, radiolabeled probe plus increasing concentrations of the unlabeled *rnpB* probe (non-specific).

Figure S4

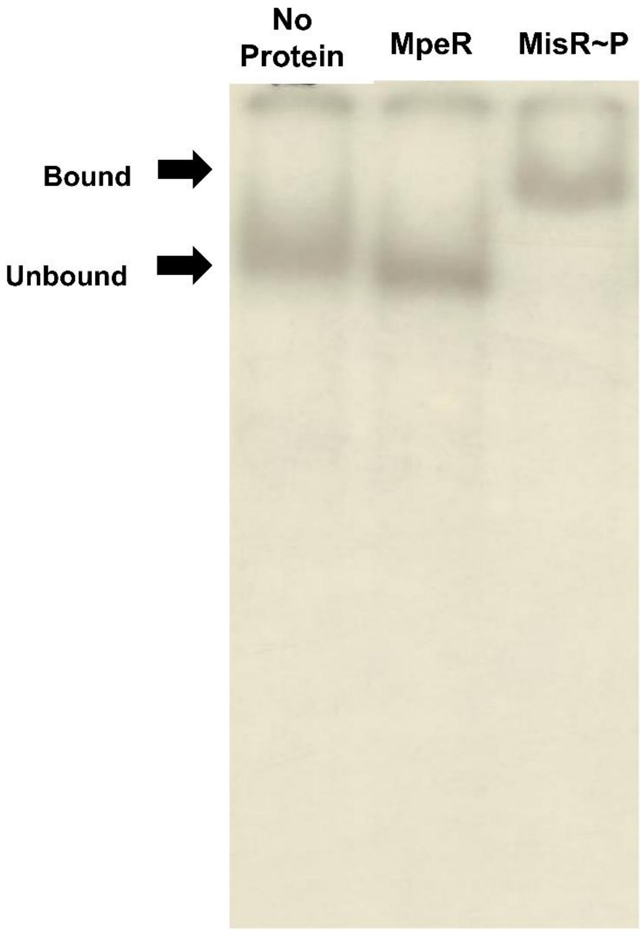

MisR and not MpeR binds to the *ompA* promoter. EMSA demonstrating direct MisR binding. The purified MisR~P protein binds to the *ompA* promoter region from strain FA19 while purified MpeR does not. The EMSA employed 5 ng of each radiolabeled probe.

Figure S5

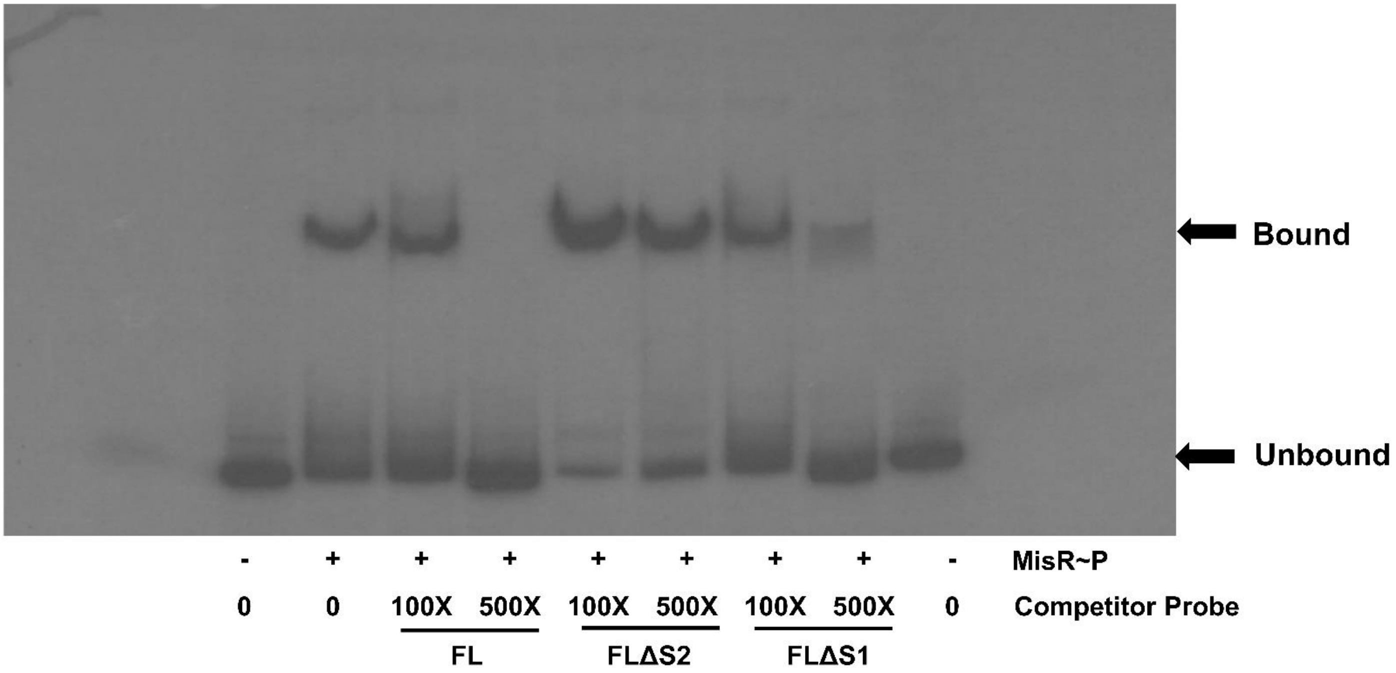

Mapping of primary MisR binding sites (Full Autoradiograph). Shown is the full-length autoradiograph for the competitive EMSA from Figure 5 demonstrating preferential MisR binding to specific sites in the promoter region. Lane 1 and 9, radiolabeled probe alone (5 ng); Lane 2, radiolabeled probe plus MisR~P (1.5 µg); Lanes 3-4, radiolabeled probe plus unlabeled FL competitor probes; Lanes 5-6 radiolabeled probe plus unlabeled FLΔS2 competitor probes; Lanes 7-8 radiolabeled probe plus unlabeled FLΔS1 competitor probes.

Figure S6

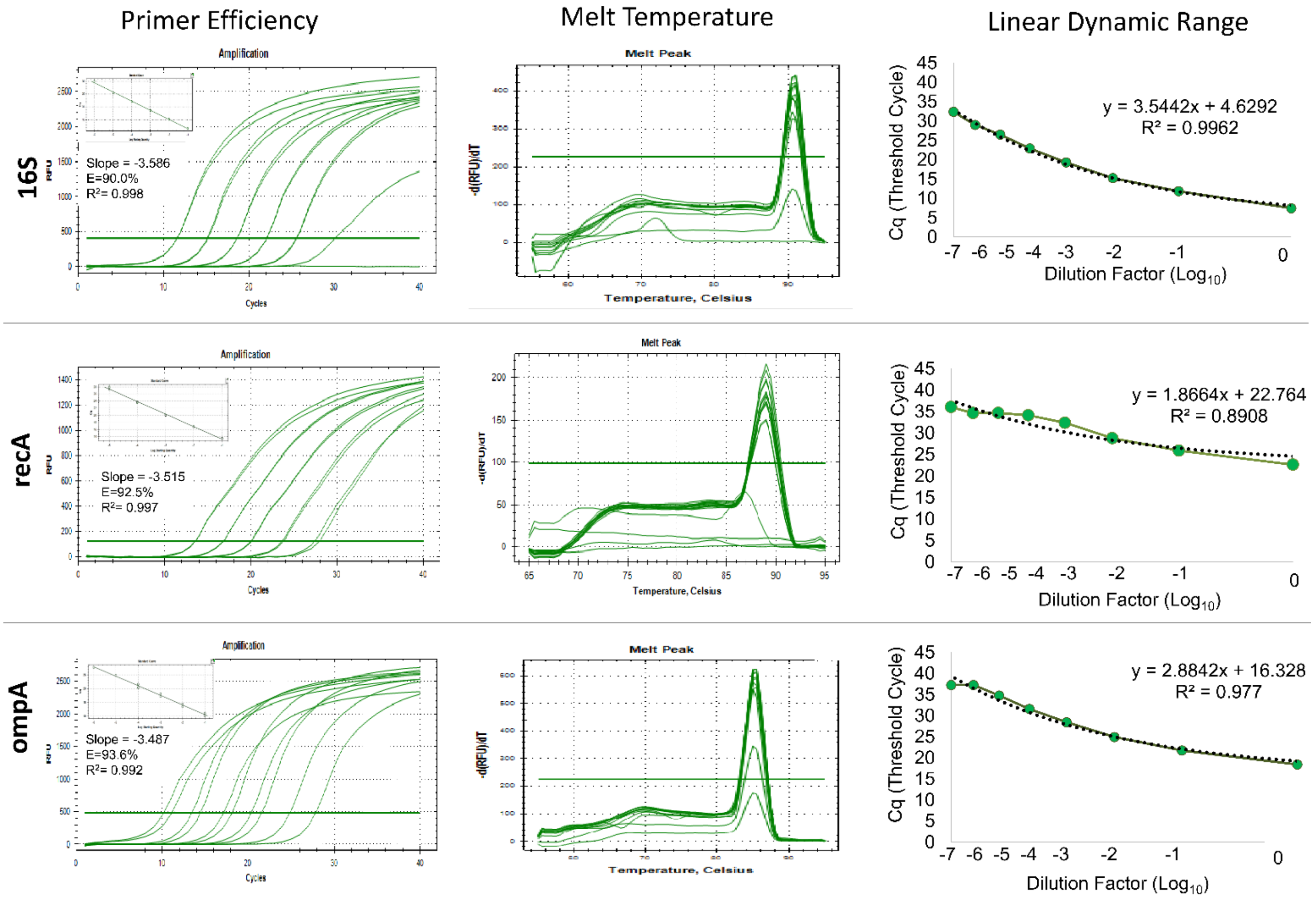

Validation of qPCR Method. The qPCR method was validated by looking at Primer Efficiency against genomic DNA; Target Specificity (Melt Temperature); and Linear Dynamic Range of the primers against cDNA. Each primer set utilized in this study (*16S, ompA* and *recA)* were validated prior to use in qRT-PCR.
